# Artificial Intelligence Applications and Self-Learning 6G Networks for Smart Cities Digital Ecosystems: Taxonomy, Challenges, and Future Directions

**DOI:** 10.3390/s22155750

**Published:** 2022-08-01

**Authors:** Leila Ismail, Rajkumar Buyya

**Affiliations:** 1 Intelligent Distributed Computing and Systems (INDUCE) Research Laboratory, Department of Computer Science and Software Engineering, College of Information Technology, United Arab Emirates University, Abu Dhabi 15551, United Arab Emirates; 2 National Water and Energy Center, United Arab Emirates University, Abu Dhabi 15551, United Arab Emirates; 3 Cloud Computing and Distributed Systems (CLOUDS) Laboratory, School of Computing and Information Systems, The University of Melbourne, Parkville, VIC 3010, Australia; rbuyya@unimelb.edu.au

**Keywords:** Artificial Intelligence (AI), beyond 5G, blockchain, Deep Learning, Internet of Things (IoT), Machine Learning, metaheuristics algorithms, Sixth Generation (6G) wireless communication, smart city

## Abstract

The recent upsurge of smart cities’ applications and their building blocks in terms of the Internet of Things (IoT), Artificial Intelligence (AI), federated and distributed learning, big data analytics, blockchain, and edge-cloud computing has urged the design of the upcoming 6G network generation, due to their stringent requirements in terms of the quality of services (QoS), availability, and dependability to satisfy a Service-Level-Agreement (SLA) for the end users. Industries and academia have started to design 6G networks and propose the use of AI in its protocols and operations. Published papers on the topic discuss either the requirements of applications via a top-down approach or the network requirements in terms of agility, performance, and energy saving using a down-top perspective. In contrast, this paper adopts a holistic outlook, considering the applications, the middleware, the underlying technologies, and the 6G network systems towards an intelligent and integrated computing, communication, coordination, and decision-making ecosystem. In particular, we discuss the temporal evolution of the wireless network generations’ development to capture the applications, middleware, and technological requirements that led to the development of the network generation systems from 1G to AI-enabled 6G and its employed self-learning models. We provide a taxonomy of the technology-enabled smart city applications’ systems and present insights into those systems for the realization of a trustworthy and efficient smart city ecosystem. We propose future research directions in 6G networks for smart city applications.

## 1. Introduction

**Figure 1:**
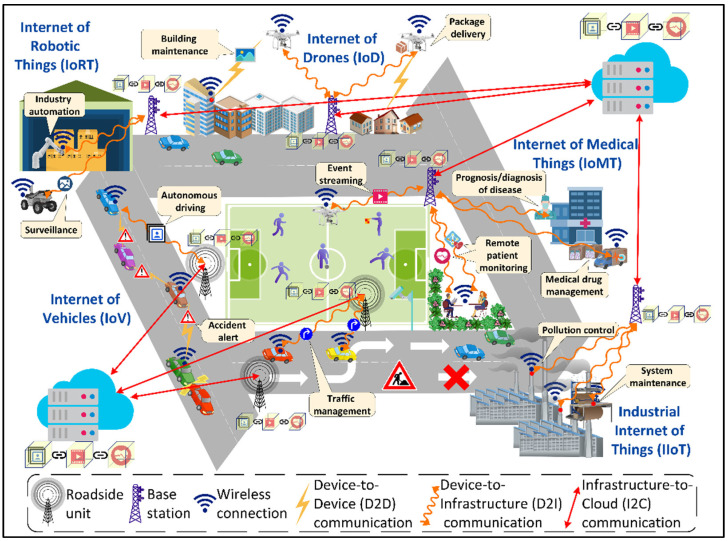
A view of smart city digital ecosystem.

With the prominence of connected smart cities and the recent emergence of a smart city’s mobile applications and their building blocks architecture in terms of Internet of Things (IoT) [1], Artificial Intelligence (AI) [2], federated and distributed learning [3], big data analytics [4], blockchain [5], and edge-cloud computing [6], the implementation of a new generation of networks has been prompted. While optimization strategies at the application level along with a fast network, such as the currently in-deployment 5G networks, play an important role, this is not enough for AI-based distributed, dynamic, contextual, and secure smart city applications enabled by emergent technologies [7]. As shown in Figure 1, these applications include, but are not limited to, autonomous driving, accident prevention and traffic management enabled by the Internet of Vehicles (IoV), remote patient monitoring, medical drug supply chain management, the prognosis/diagnosis of diseases empowered by the Internet of Medical Things (IoMT), industry automation and surveillance using the Internet of Robotic Things (IoRT), building maintenance and package delivery enabled by the Internet of Drones (IoD), system maintenance and pollution control enabled by the Industrial Internet of Things (IIoT), interactive gaming and aerospace navigation using Holographic Communication (HC), immersive training and guided repair enabled by Extended Reality (XR), intelligent transportation systems and smart connected healthcare using blockchain, and data analytics empowered by edge-cloud computing. These applications and their rigorous support for meeting requirements in terms of the quality of services (QoS) and dependability to satisfy the Service-Level-Agreement (SLA) for the end users [8,9,10,11] have been a driving force for the evolution of networks.

The energy consumption of the smart cities’ digital ecosystem serving these applications is a major issue causing environmental threats and increasing electricity bills, requiring immediate sustainable remedies [12]. Estimates show that cloud data centers, considered the backbone of smart cities, will be responsible for 4.5% of the total global energy consumption by 2025 [13]. The average electricity cost for powering a data center could be as high as $3 million per year [14]. Furthermore, it is predicted that by 2040, Information and Communications Technology (ICT) will be responsible for 14% of global carbon emissions [15]. Consequently, the underlying communication networks should focus on deploying efficient, dependable, and secure applications in smart city applications while considering the critical requirements of privacy, energy efficiency, high data rates, and ultra-low latencies for those applications.

Therefore, industries and academia have started to look beyond 5G networks and design the upcoming 6G. In particular, 6G network designers propose the use of AI in its underlying protocols and operations for optimal performance and energy efficiency. The vibe over AI and its tremendous potential for intelligent applications and network systems, in combination with IoT smart city applications, have been a great motivation in developing AI-IoT-based solutions. These solutions require huge communication and computation resources, giving rise to latency, energy consumption, network congestion, and privacy leakage.

The current research on this topic either focuses on the requirements of AI applications in a smart city ecosystem that can benefit from the underlying 6G networks or on the self-learning 6G networks for agility, flexibility, and energy efficiency. To our knowledge, no work adopts a holistic perspective considering the underlying 6G networks, middleware, and technology-enabled applications for an intelligent and integrated smart city digital ecosystem.

The main contributions of our paper are as follows.

1. We provide a temporal evolution of the wireless communication network generations from 1G to AI-enabled 6G and capture the inherent challenges and technological requirements that lead to the development of a given network generation over a certain period.
2. We present self-learning models that would be infused in 6G to accommodate the strict requirements of smart city applications in terms of low latency, high reliability, security, energy efficiency, execution time, and context awareness.
3. We propose a taxonomy of distributed, dynamic, and contextual AI applications in 6G networks based on the underlying technology used by those applications. In addition, we provide insights on the requirements of these applications that should be considered by the underlying 6G networks.
4. We propose future directions toward the realization of a trustworthy and efficient digital ecosystem consisting of intelligent and connected applications, the middleware, the underlying technologies, and the 6G network systems.

The rest of the paper is organized as follows. Section 2 provides a categorization and overview of related surveys. The temporal evolution of wireless communication network generations is presented in Section 3. Section 4 and Section 5 synthesize the taxonomies of AI-enabled 6G networks with their self-learning models and technology-enabled smart city applications in 6G. Future research directions are discussed in Section 6. Section 7 summarizes and concludes the paper.

## 2. Related Survey

There have been few surveys on AI-enabled applications and AI-6G in smart cities. We classify these surveys into two categories based on the survey’s approach: (1) a top-down approach highlighting the requirements of AI applications in terms of networks’ capabilities [16,17] and (2) a down-top perspective focusing on AI-enabled 6G networks for agile, flexible, and efficient systems [18,19,20,21].

Concerning the top-down approach, Akhtar et al. [16] presented the projected 6G architecture and its characteristics along with potential technologies enabling the envisioned network generation systems. The authors focused on quantum communication and Machine Learning, blockchain, tactile internet, and free duplexing and spectrum sharing technologies. Furthermore, the authors discussed e-heath and bio-sensing, HC, and IoT applications that will be underlined by 6G networks. However, the authors did not analyze the requirements of the technology-enabled applications in 6G. Using a similar approach, Tataria et al. [17] explained the 6G networks architecture, characteristics, and deployment scenarios. In addition, the authors analyzed the requirements for applications in 6G systems enabled by HC, tactile and haptic internet, edge-cloud computing, and IoT technologies. However, these works [16,17] do not focus on employing self-learning models on the underlined 6G networks layer for security, agility, flexibility, and energy efficiency.

Regarding the down-top approach, Yang et al. [18] proposed an AI-enabled 6G architecture for radio network resource management and service provisioning. Similarly, Letaief et al. [19,20] analyzed the potential of AI for 6G networks design and optimization. Zhang and Zu [21] presented a survey on AI-enabled 6G networks for radio interface, intelligent traffic control, resource management, performance and energy optimization, and security. However, these works do not analyze the requirements of AI applications in 6G networks.

Table 1 summarizes the related survey and their comparison with our work. In contrast to these surveys based on top-down or down-top approaches, in this paper, the 6G technology is prospected from a holistic perspective, where self-learning models are, respectively, inserted into the main technical layers of 6G to achieve the requirements of building an integrated smart city digital ecosystem as a whole, not just looking at application needs or network requirements. In this approach, in addition to AI-enabled 6G network systems and employed self-learning models, middleware and technology-enabled applications and their requirements for an intelligent and connected contextual computing and communication smart cities ecosystem are analyzed.

## 3. Evolution of Wireless Communication Technology (1G–6G)

**Figure 2:**
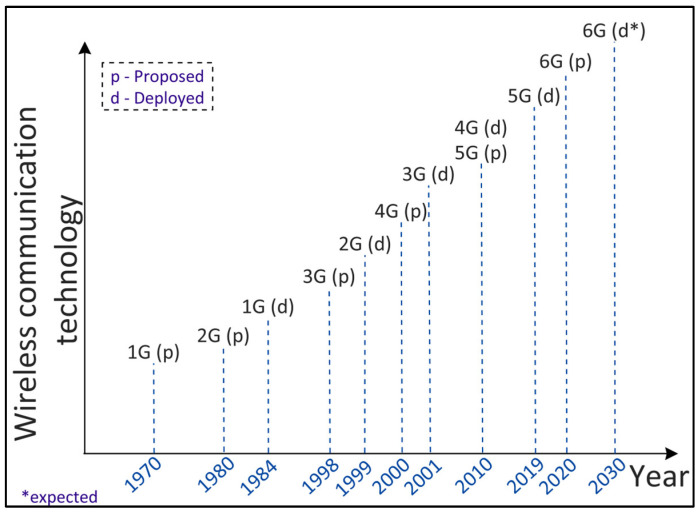
Evolution of wireless communication technology from 1G to 6G.

Wireless communication technology has evolved over the years intending to provide high-speed, reliable, and secure communication. Figure 2 shows the evolution of wireless network development from 1G to 6G, including the year of proposing and that of deploying a particular network generation over time. In the following, we explain each evolution along with its applications and shortcomings.

### 3.1. First Generation (1G) Technology

The first generation (1G) communication system was introduced in 1978 in the United States based on the Advanced Mobile Phone System (AMPS) [22]. The AMPS is an analog cellular system allocated with 50MHz bandwidth with a frequency range of 824–894 MHz [23]. The bandwidth in the AMPS is divided into sub-channels of 30 KHz, each using Frequency Division Multiple Access (FDMA) for multiple users to send data. In 1979, 1G was commercially launched in Japan by the Nippon Telegraph and Telephone (NTT) DoCoMo Company. In 1981, the Nordic Mobile Telephone (NMT) standard for 1G was developed by the Nordic countries such as Norway, Denmark, Switzerland, Finland, and Sweden. In 1983, the AMPS was commercially launched in the United States and was later used in Australia. The Total Access Communication Systems (TACS) standard was introduced in the United Kingdom for 1G [24]. First generation technology supported voice calls with up to 2.4 Kbps of bandwidth within one country. However, the underlying technology could not handle international voice and conference calls, and other applications such as messaging services, emails, and accessing information over a mobile wireless network. In addition, being an analog system, 1G suffered from bad voice quality and poor handoff reliability. Furthermore, 1G was less secure. To overcome these shortcomings, the 2G network generation was introduced.

### 3.2. Second Generation (2G) Technology

To enable applications such as international voice calls, messaging, and access to information over a wireless network, which require a high data transfer rate, and to make communication more secure, the second-generation (2G) wireless technology was designed in the 1980s and introduced in 1991 under the Global System for Mobile (GSM) communication standards in Finland [25]. The analog system of 1G was replaced by a digital system enabling the encryption of voice calls and thus providing security. The GSM uses Time Division Multiple Access (TDMA) such that each network user is allocated the channel bandwidth based on time slots [26]. The GSM operates on a 900–1800 MHz frequency band except for in America where it operates in the 1900 MHz band. TDMA was later used by other digital standards such as the Digital AMPS (D-AMPS) in the United States and the Personal Digital Cellular (PDC) in Japan. As an alternative to TDMA, Code Division Multiple Access (CDMA) was introduced in the United States on the IS-95 standard [22] which allowed multiple network users to simultaneously transmit data based on assigned unique code sequences. In addition to international roaming voice calls, 2G supported conference calls, call hold facility, short message services (SMS), and multimedia message services (MMS) with a data rate up to 9.6 Kbps.

The continuous evolution of the GSM technology led to the development of General Packet Radio Service (GPRS), referred to as 2.5 G, which implemented packet switching in addition to circuit switching. GPRS has provided additional services such as Wireless Application Protocol (WAP) access and internet communication such as e-mail and World Wide Web (WWW) access [27]. It provides data rates up to 115 Kbps [28]. GPRS further evolved to the Enhanced Data Rates for GSM evolution (EDGE), providing higher data rates. For instance, a 40 KB text file can be transferred in 2 s using EDGE compared to 6 s in GPRS. EDGE was deployed on GSM networks in 2003 by Cingular (now AT&T) in the United States. The peak data speed of 2G is 50 Kbps using GPRS and 1 Mbps using EDGE. However, 2G networks were not capable of handling video conferencing, navigation services, and other applications which require high data rates, leading to the 3G network generation.

### 3.3. Third Generation (3G) Technology

The Third Generation Partnership Project (3GPP) was formed in 1998 to provide a standardized frequency across the globe for mobile networking, enabling high data rate services such as video calls, navigation, and interactive gaming. It is based on the International Mobile Telephone (IMT)-2000 standard and was first made available in Japan by the NTT DoCoMo in 2001. The IMT-2000 focused on providing a wider coverage area, improving the QoS, and making services available to users irrespective of their location [29]. One of the requirements of the IMT-2000 was to have a minimum speed of 200 Kbps for a network to be 3G. The third generation has introduced wireless technology such as video conferencing and video downloading with an increased data transmission rate at a lower cost. It increased the efficiency of the frequency spectrum by improving the audio compression during a call, allowing more simultaneous calls in the same frequency range. Third generation technology evolved between 2000 and 2010 to provide Universal Mobile Telecommunications System (UMTS)-based networks with higher data rates and capacities. In particular, High-speed Downlink Packet Access (HSDPA) was deployed (also referred to as 3.5G), which is a packet-based data service providing downlink data rates 8–10 Mbps. To provide services for applications that require high data rates such as interactive gaming, High-Speed Uplink Packet Access (HSUPA) was introduced (referred to as 3.75G) which enabled an uplink data transmission speed of 1.4–5.8 Mbps. However, IP telephony, 3D videos, and High Definition (HD) mobile TV were not supported by the 3G technology, leading to the foundation of the 4G network generation.

### 3.4. Fourth Generation (4G) Technology

To transmit data, voice, multimedia, and internet services at a higher rate, quality, and security at a low cost, the Fourth Generation (4G) was initiated in the late 2000s as an all-IP-based network system. Fourth Generation technology was first used commercially in Norway in 2009 after its successful field trial in Japan in 2005. It aimed to provide peak data rates of up to 1 Gbps at low mobility and 100 Mbps at high mobility and is based on Long-Term Evolution (LTE) and Wireless Interoperability for Microwave Access (WiMAX) technologies. The LTE standard was further enhanced to LE-Advanced Pro (referred to as 4.5G) to increase the mobile broadband and connectivity performances [22]. However, 4G was not capable of operating applications that require image processing, such as machine vision, smart connected cars, and augmented reality, giving rise to the 5G network generation.

### 3.5. Fifth Generation (5G) Technology

To obtain a consistent QoS, low end-to-end latency, reduced cost, and massive device connectivity, the Fifth Generation (5G) communication technology was established to support applications such as AR, home and industrial automation, and machine vision. Fifth Generation technology was first offered in South Korea in 2019. It provides a data rate of 20 Gbps in the downlink and 10 Gbps in the uplink, and is aimed to support three generic services; Enhanced Mobile Broadband (eMBB), Massive Machine-type Communications (mMTC), and Ultra-Reliable Low-Latency Communications (URLLCs) [30]. eMBB aims to deliver peak download speeds of over 10 Gbps to support applications such as Ultra-High Definition (UHD) videos and AR. mMTC defines the requirement to support one million low-powered economical devices per Km^2^ with a battery life of up to 10 years. It can support applications such as smart homes and industrial automation that involve several sensors, controllers, and actuators. URLLC sets the requirement of high reliability (99.99%), extremely low latencies (<1 ms), and support for low data rates (bps/Kbps) to support applications such as social messaging services, traffic lights, self-driving cars, and smart healthcare. In 2020, Huawei, as part of the 5.5G vision, proposed three additional sets of services; Uplink-centric Broadband Communication (UCBC), Real-Time Broadband Communication (RTBC), and Harmonized Communication and Sensing (HCS) [31]. UCBC aims to increase the uplink bandwidth by 10-fold to support applications involving machine vision. For Augmented Reality (AR), Virtual Reality (VR), and Extended Reality (XR) applications, RTBC would provision large bandwidth and low latency services with a certain level of reliability. HCS focuses to offer communication and sensing functionalities for connected cars and drone scenarios. However, with the emergence of an interactive and connected IoT, communicating IoV, and holographic applications, the 5G networks could not manage the stringent high computing and communications requirements of those applications. Consequently, the 6G network generation was instigated.

### 3.6. Sixth Generation (6G) Technology

The Sixth Generation (6G) technology was envisioned in 2019–2020 to transform the “Internet of Everything” into an “Intelligent Internet of Everything” with more stringent requirements in terms of a high data rate, high energy efficiency, massive low-latency control, high reliability, connected intelligence with Machine Learning (ML) and Deep Learning (DL), and very broad frequency bands [19]. Three new application services are proposed; Computation Oriented Communications (COC), Contextually Agile eMBB Communications (CAeC), and Event-Defined uRLLC (EDuRLLC) [19]. COC will enable the flexible selection of resources from the rate-latency-reliability space depending on the available communication resources to achieve a certain level of computational accuracy for learning approaches. CAeC will provision eMBB services that would be adaptive to the network congestion, traffic, topology, users’ mobility, and social networking context. 6G-EDuRLLC targets the 5G-URLLC applications that will operate in emergency or extreme situations having spatial-temporal device densities, traffic patterns, and infrastructure availability.

Table 2 summarizes the characteristics of the wireless communication networks from 1G to 6G [16,32].

## 4. Artificial Intelligence (AI)-Enabled 6G Networks

**Figure 3:**
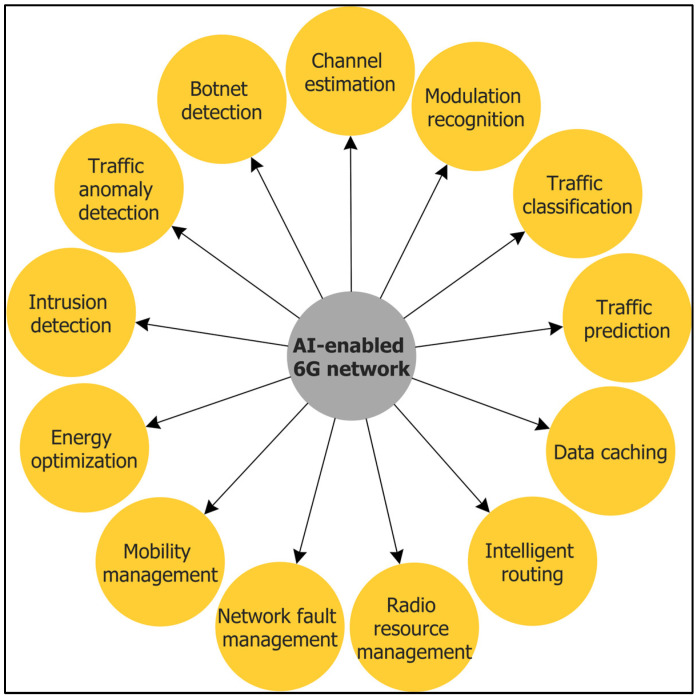
Artificial Intelligence (AI)-enabled 6G networks.

The disruptive emergence of highly distributed smart city mobile applications [33,34,35] such as the IoV, IoMTs, IoD, IoRT, IIoT, 3D virtual reality, and their stringent requirements in terms of the QoS and the need for the service providers to satisfy SLAs, have been a driving force for 6G. In addition, many of those applications are AI-Big data-driven, making it challenging if not impossible for 5G to satisfy those requirements. Therefore, 6G must provide battery-free device capabilities, very high data rates, a very high energy efficiency, massive low-latency control, very broad frequency bands, and ubiquitous broadband-global network coverage beyond what 5G LTE can offer. To achieve that level of efficiency, in contrast to 5G, 6G needs to be equipped with context-aware algorithms to optimize its architecture, protocols, and operations. For this purpose, 6G will infuse connected intelligence in its design in an integrating communication, computing, and storage infrastructure from the edges to the cloud and core infrastructure. Supporting a wide range of applications that are demanding in terms of low latency, high reliability, security, and execution time requires an AI-enabled optimization for 6G [19]. Traditional approaches using statistical analysis based on prior knowledge and experiences via the deployment of the Software Defined Network (SDN) [19] will not be any more effective due to the elapsed time from analysis to decision making. Consequently, ML and DL algorithms are used to solve several issues in networking, such as caching and data offloading [36]. In this section, we present AI-enabled 6G network protocols and mechanisms (Figure 3) and their employed self-learning ML/DL models.

### 4.1. Channel Estimation

**Figure 4:**
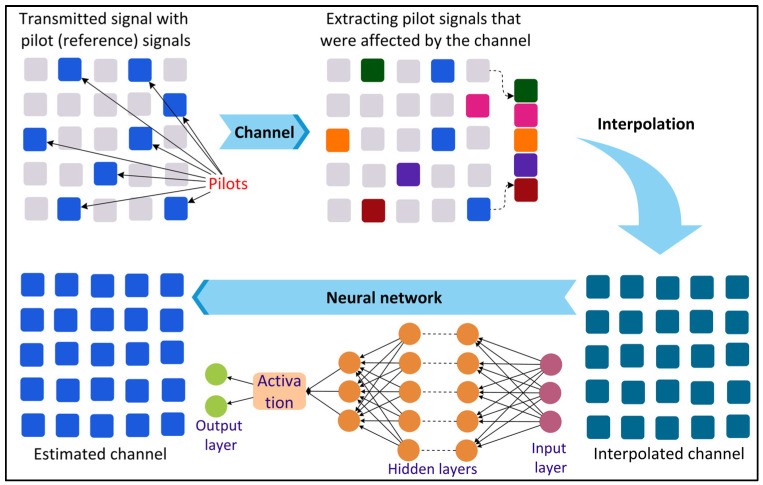
Deep Learning (DL)-based channel estimation.

To fulfill the demanding requirements of smart city applications in terms of high data rate (Tbps), low latency (order of 0.1–1 ms), and high reliability (order of 10^−9^) [37], the 6G radio access will be enabled by emerging technologies such as Terahertz communication [38], visible-light communication, ultra-massive multiple-input multiple-output (MIMO) [39], and large intelligent surfaces [40]. These technologies will increase the complexity of the radio communication channels, making efficient channel estimation challenging using the traditional mathematical approaches. The wireless communication channel attenuates the phase shifts and attenuates and adds noise to the transmitted information. In this context, channel estimation can be defined as the process of estimating the characteristics of the communication channel to recover the transmitted information from the channel effect. To increase the performance and capacity of 6G communication, precise and real-time channel estimation becomes crucial. Recently, DL has gained wide attention for precise channel estimation. Figure 4 shows a DL-based channel estimation process where the signal is first transmitted along with some pilot (reference) signals. The effects of the channel on the pilot signals are then extracted. The channel characteristics are then estimated by a DL method using the interpolated channel.

Ye et al. [41] proposed a Deep Neural Network (DNN)-based approach for channel estimation and symbol detection in an Orthogonal Frequency Division Multiplexing (OFDM) system. The DNN model is trained offline using OFDM samples generated using different information sequences under distinct channel conditions. The model is then used to recover the transmitted information without estimating the channel characteristics. Gao et al. [42] proposed a Convolutional Neural Network (CNN)-based channel estimation framework for massive MIMO systems. The authors used one-dimensional convolution to shape the input data. Each convolutional block consists of a batch normalization layer to avoid gradient explosions [43] and a Rectified Linear Unit (Relu) activation function.

### 4.2. Modulation Recognition

**Figure 5:**
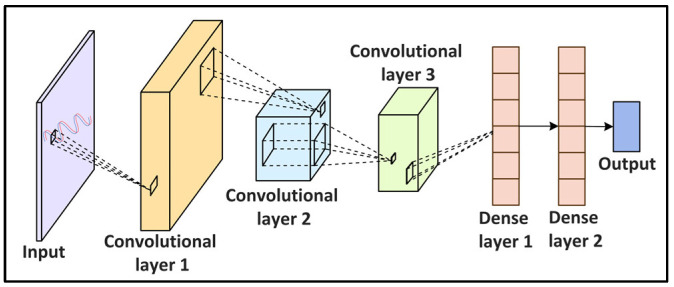
Convolutional Neural Network (CNN)-based modulation recognition in networking.

With the increasing data traffic in smart cities, different modulation methods are employed in a communication system for efficient and effective data transmission by modulating the transmitted signal. In this context, modulation recognition aims to identify the modulation information of the signals under a noisy interference environment [44]. Modulation recognition aids in signal demodulation and decoding for applications such as interference identification, spectrum monitoring, cognitive radio, threat assessment, and signal recognition. The conventional decision-theory-based and statistical-pattern-recognition-based methods for modulation recognition become computationally expensive and time consuming for smart city applications [44]. DL can be used as an alternative to improve the accuracy and efficiency of modulation recognition as shown in Figure 5. Zhang et al. [44] investigated the applicability of a CNN and Long Short-Term Memory (LSTM) for modulation recognition as the former is good for the automatic feature extraction of spatial data and the latter performs well for sequential data. Yang et al. [45] proposed the use of CNN and Recurrent Neural Networks (RNNs) for modulation recognition over additive white Gaussian noise and Rayleigh fading channels. The authors found that DL algorithms perform modulation recognition more accurately compared to ML algorithms such as the Support Vector Machine (SVM). To ensure the privacy and security of the transmitted data, Shi et al. [46] proposed a CNN-based federated learning approach with differential privacy for modulation recognition.

### 4.3. Traffic Classification

**Figure 6:**
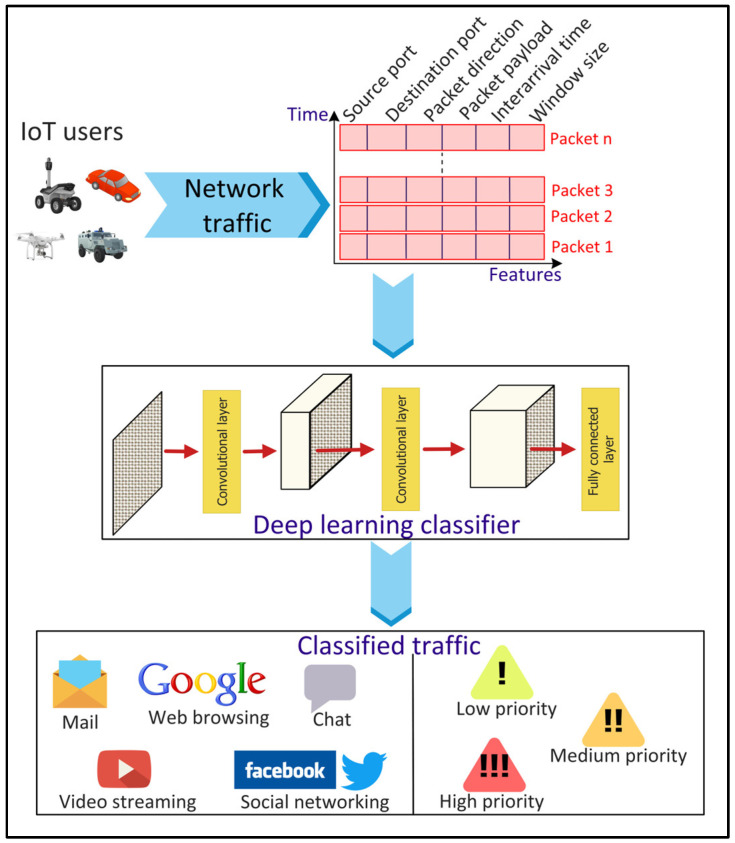
Deep Learning (DL)-based network traffic classification for Internet of Things (IoT) applications.

The categorization of network traffic into different classes, referred to as traffic classification, is important to ensure the QoS, control pricing, resource management, and security of smart city applications. The simplest method for traffic classification involves mapping the applications’ traffic to port numbers [47]. However, this technique provides an inaccurate classification since several applications use dynamic port numbers. Payload-based methods are alternatives to the port-based techniques where the traffic is classified by examining the packet payload [47]. However, the traffic payload cannot be accessed in scenarios where the packets are encrypted due to privacy and security concerns. Consequently, ML/DL-based methods can be used to address the issues of the conventional methods (Figure 6). Ren, Gu, and Wei [48] proposed a Tree-RNN to classify network traffic into 12 different classes. The proposed DL model consists of a tree structure that divides the large classification problem into smaller ones, with each class represented by a tree node. Lopez-Martin et al. [49] proposed a hybrid RNN- and CNN-based network to classify traffic from IoT devices and services. CNN layers extract complex network traffic features automatically from the input data, eliminating the feature selection process used in the classical ML approaches.

### 4.4. Traffic Prediction

**Figure 7:**
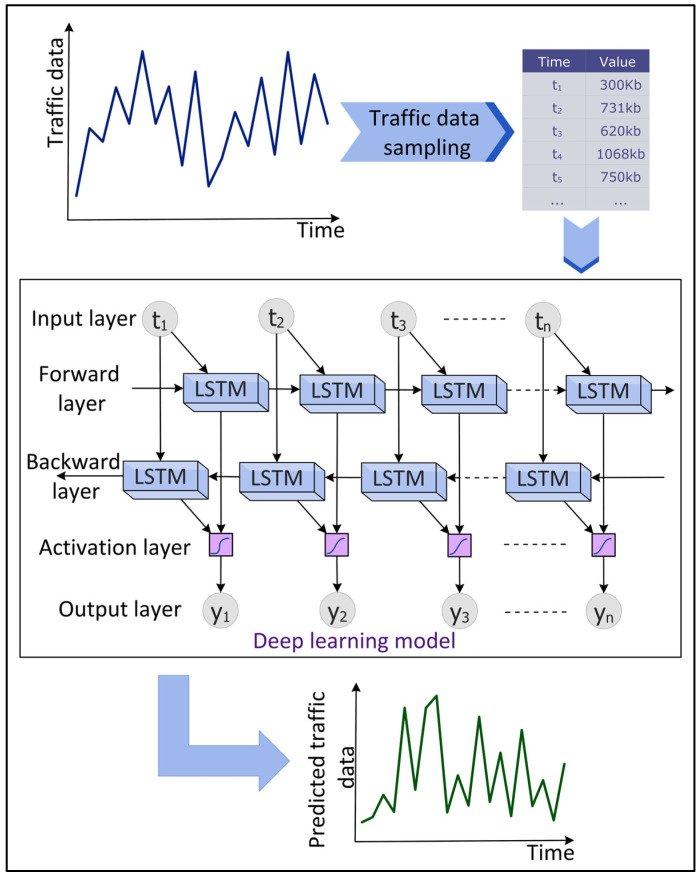
Deep Learning (DL)-based time series prediction of network traffic data.

Network traffic predictions focus on predicting future traffic using previous traffic data. This aids in proactively managing the network and computing resources, improving the QoS, making the network operations cost-effective, and detecting anomalies in the data traffic. DL has shown potential in predicting network traffic accurately in real time. Figure 7 shows an overview of the DL-based predictions of the network traffic data. Vinayakumar et al. [50] evaluated the performance of different RNNs, namely the simple RNN, Long Short-Term Memory (LSTM), Gated Recurrent Unit (GRU), Identity Recurrent Unit (IRNN), and Feed-forward Neural Network (FNN) to predict network traffic using a GEANT backbone networks dataset. The experimental results showed that LSTM predicts the network traffic with the least MSE. Aloraifan, Ahmad, and Alrashed [51] used Bi-directional LSTM (Bi-LSTM) and Bi-directional GRU (Bi-GRU) to predict the network traffic matrix. To increase the prediction accuracy, the authors combined a CNN with Bi-LSTM or Bi-GRU. The authors found that the prediction performance of DL algorithms depends on the configuration of the neural network parameters.

### 4.5. Data Caching

**Figure 8:**
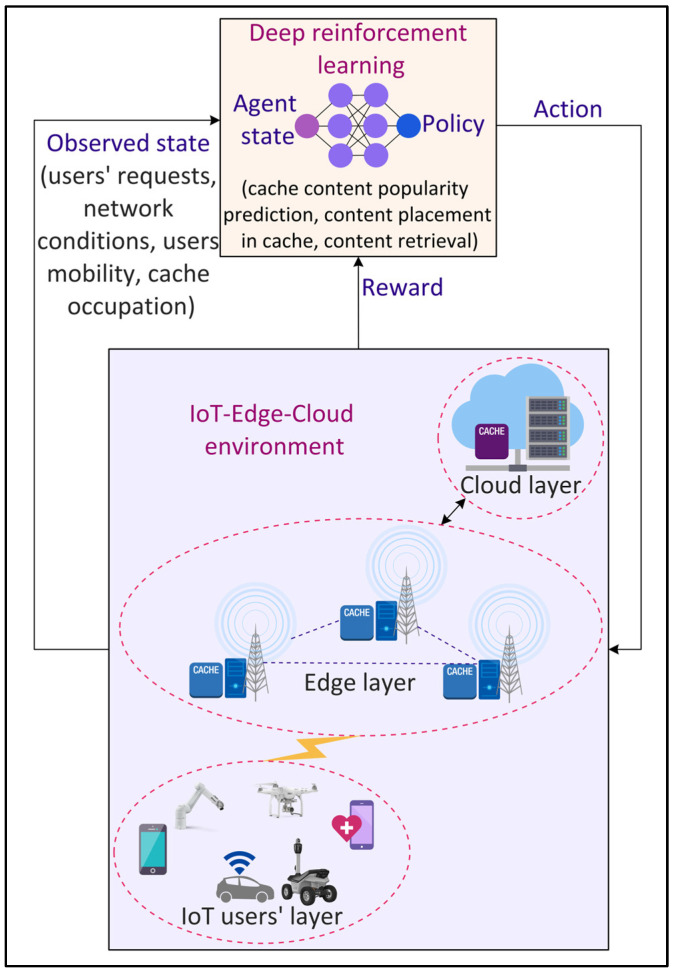
Deep Reinforcement Learning (DRL)-based data caching in the Internet of Things (IoT) environment.

Internet data traffic is exploding at a rapid pace with the increasing popularity and demand of different smart city applications such as infotainment, AV, VR, interactive gaming, and XR. Consequently, it becomes challenging to accommodate these data in terms of storage and transmission for applications that require an ultra-low latency such as autonomous vehicles and smart healthcare. To address this challenge, edge caching [52] is seen to be a potential solution that provides storage facilities to the IoT data at the edge of the network, i.e., in proximity to the mobile devices. This enables IoT applications to retrieve data in real time from the edge resources, eliminating backhaul link communication. Consequently, edge caching reduces data transmission time and energy. However, determining the optimal cache content and cache placement strategies in a dynamic network is challenging [53]. DL can be effective in designing optimal caching strategies, as shown in Figure 8. Jiang et al. [54] proposed a distributed deep Q-learning-based caching mechanism to improve the edge caching efficiency in terms of cache hit rate. The mechanism involves the prediction of users’ preferences offline followed by the online prediction of content popularity. However, due to the limited caching resources of the edge nodes and spatial-temporal content demands from the mobile users, cooperative edge caching schemes are required. Zhang et al. [55] proposed a DRL-based cooperative edge caching approach that enables the communication between distributed edge servers to enlarge the size of the cache data. However, cooperative schemes often collect and analyze the data at a centralized server. The sharing of sensitive data, such as users’ preferences and content popularity, among different edge and cloud servers, raises privacy concerns. To tackle this challenge, federated learning (FL) can be a promising solution in which learning models to predict content popularity are trained locally at the IoT devices for cooperative caching [55].

### 4.6. Intelligent Routing

**Figure 9:**
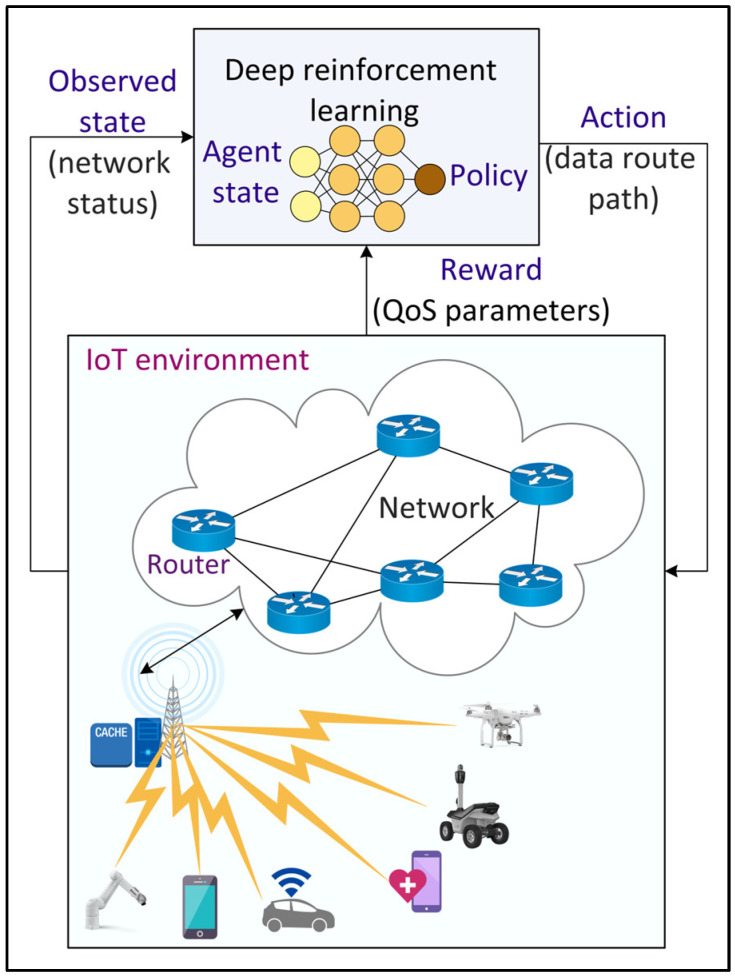
Deep Reinforcement Learning (DRL)-based intelligent routing in the Internet of Things (IoT) environment.

To manage the network traffic efficiently and to fulfill the QoS requirements of 6G applications, several routing strategies have been developed. However, the traditional routing protocols developed using meta-heuristic approaches become computationally expensive with increasing traffic variability. Consequently, ML and DL-based approaches have been proposed to address the shortcomings of the traditional methods. Figure 9 shows an example of DL-based intelligent routing in an IoT environment. Tang et al. [56] proposed a real-time deep CNN-based routing algorithm in a wireless mesh network backbone. A CNN model with two convolutional layers and two fully connected layers is trained periodically using the continuous stream of network data. Liu et al. [57] proposed a DRL-based routing in software-defined data center networks by recombining network resources (such as cache and bandwidth) based on their effectiveness in reducing delay and then using DRL for routing with the recombined state. The employed DNN model within DRL consists of two fully connected hidden layers with 30 neurons each. The CNN model in the actor and critic networks of DRL consists of two max pooling layers, three convolutional layers with eight filters, and one fully connected layer with 30 neurons. A Relu activation function is employed in all the layers.

### 4.7. Radio Resource Management

With the future 6G networks, the density of small-cell networks increases drastically. Consequently, radio resource management has emerged for the system-level management of co-channel interference, radio resources, and other radio transmission characteristics in a wireless communication system to utilize the radio spectrum efficiently. With the increasing dynamicity and complexity of network generations towards 6G, the traditional heuristic-based approaches for radio resource management become inaccurate. ML/DL-based approaches are explored as an alternative solution. Shen et al. [58] proposed graph neural networks for radio resource management in a large-scale network by modeling the wireless network as a wireless channel graph and then formulating the resource management as a graph optimization problem. The neural network consisted of three layers, an adam optimizer, and a learning rate of 0.001. Zhang et al. [59] proposed a DNN framework for radio resource management to minimize the energy consumption of the network constrained by power limitation, inference limitation, and the QoS. A DNN model with three layers, 800 neurons per layer, a 0.01 learning rate, and an adam optimizer is employed for a power optimization scheme, whereas a DNN model with four layers, 80 neurons per layer, a 0.05 learning rate, and an RMSProp optimizer is used for sub-channel allocation.

### 4.8. Network Fault Management

In network management, fault management is to detect, predict, and eliminate malfunctions in the communication network. The integration of newly emerging technologies and paradigms in 6G networks makes the network more complex, heterogeneous, and dynamic. Consequently, fault management becomes more challenging in 6G networks. ML/DL approaches have been studied recently for efficient fault management. Regin, Rajest, and Singh [60] proposed a Naïve Bayes and CNN-based algorithm for fault detection over a wireless sensor network in a distributed manner. The results show that the proposed approach accurately detects faults and is energy efficient. Regarding fault diagnosis, [61] implemented an FNN for fault detection and classification in wireless sensor networks. The DL model was tuned using a hybrid gravitational search and particle swarm optimization algorithm. Kumar et al. [62] studied the feasibility of ML and DL approaches for fault prediction on a cellular network. The results showed that a DNN with an autoencoder (AE) predicts the network fault with the highest accuracy compared to autoregressive neural networks and the SVM.

### 4.9. Mobility Management

**Figure 10:**
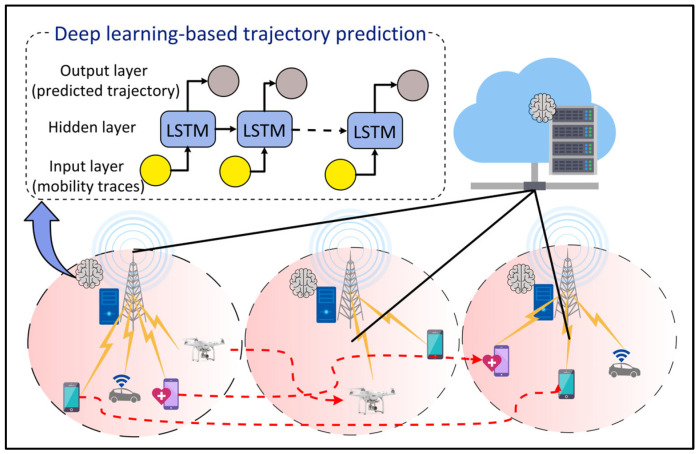
Deep Learning (DL)-based mobility management for Internet of Things (IoT) devices and users.

Sixth Generation networks will serve a spectrum of mobile applications such as the IoV, IoRT, and IoMT that require low latency and highly reliable services. To guarantee the QoS for these applications while improving the resource utilization and network bottleneck, it becomes crucial to learn and predict users’ movements. DL-based approaches can be an alternative solution, as shown in Figure 10. Zhao et al. [63] proposed a mobile user trajectory prediction algorithm by combining LSTM with RL. LSTM is used to predict the trajectories of mobile users, whereas RL is used to improve the model training time of LSTM by finding the most accurate neural network architecture for the given problem without human intervention. An initial learning rate of 0.002 is selected for LSTM and a Q-learning rate and discount factor of 0.001 and 1 are used for RL. Klus et al. [64] proposed ANN models for cell-level and beam-level mobility management optimization in the wireless network. The results showed that DL-based approaches outperform the conventional 3GPP approach for mobility management.

### 4.10. Energy Optimization

With 6G networks providing efficient connectivity to a wide range of IoT applications, the number of IoT devices is expected to increase dramatically. The data transfer, storage, and analysis from these devices will increase the energy consumption of the network. Recently, ML/DL approaches have shown potential for saving energy in wireless networks. Wei et al. [65] proposed actor-critic RL for users’ requests scheduling and resource allocation in heterogeneous cellular networks to minimize the energy consumption of the overall network. Continuous stochastic actions are generated by the actor part using a Gaussian distribution. The critic part estimates the performance of the policy and aids the actor in learning the gradient of the policy using compatible function approximation. Kong and Panaitopol [66] proposed an online RL algorithm to dynamically activate and deactivate the resources at the base station depending on the network traffic. The online RL algorithm eliminates the need for a separate model training process.

### 4.11. Intrusion Detection

**Figure 11:**
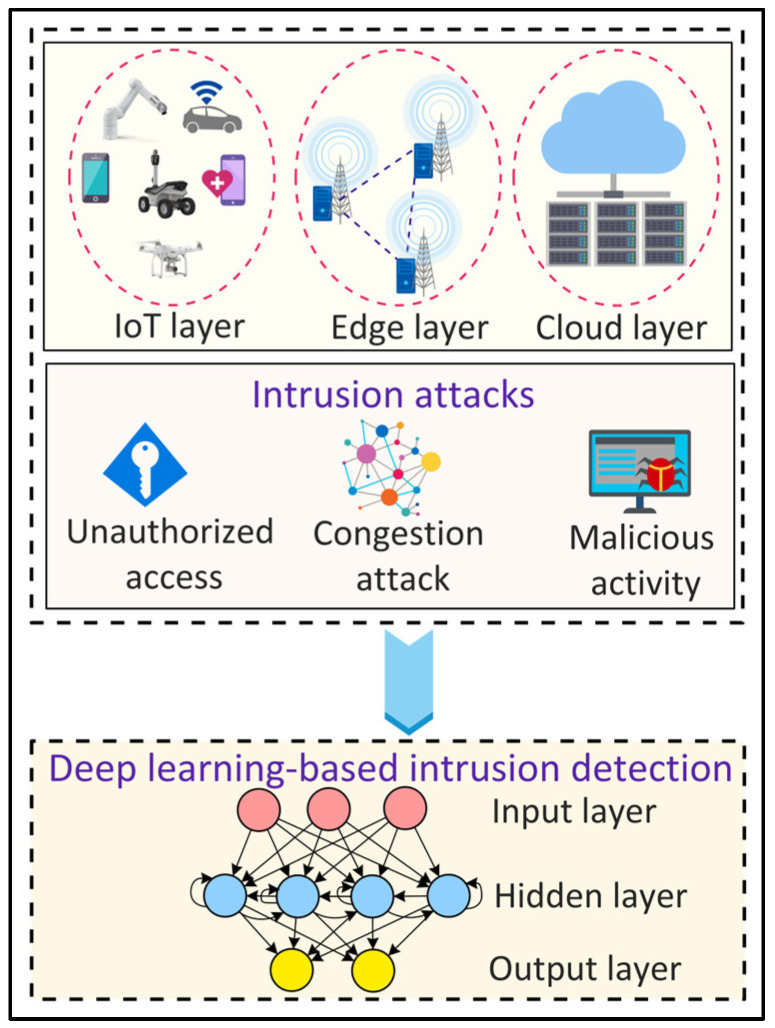
Deep Learning (DL)-based intrusion detection in Internet of Things (IoT) environments.

The evolving smart city applications running on the underlying 6G networks require high reliability and high security. In this context, intrusion detection can be used to identify unauthorized access and malicious activities in smart city applications. Figure 11 shows a DL-based approach for intrusion detection in the IoT environment. Sharifi et al. [67] proposed an intrusion detection system using a combined K Nearest Neighbor (Knn) and K-means algorithm. The proposed system employs principal component analysis for feature extraction and then uses a K-means algorithm to cluster the data. The clustered data is then classified using KNN. Yin et al. [68] proposed an RNN-based approach for binary and multi-class intrusion detection. For binary class intrusion detection, an RNN model with 80 hidden nodes and a learning rate of 0.1 provides the highest accuracy. For multi-class intrusion detection, an RNN model with 80 hidden nodes and a 0.5 learning rate yields the highest accuracy. The results show that DL approaches are better than ML approaches for intrusion detection.

### 4.12. Traffic Anomaly Detection

**Figure 12:**
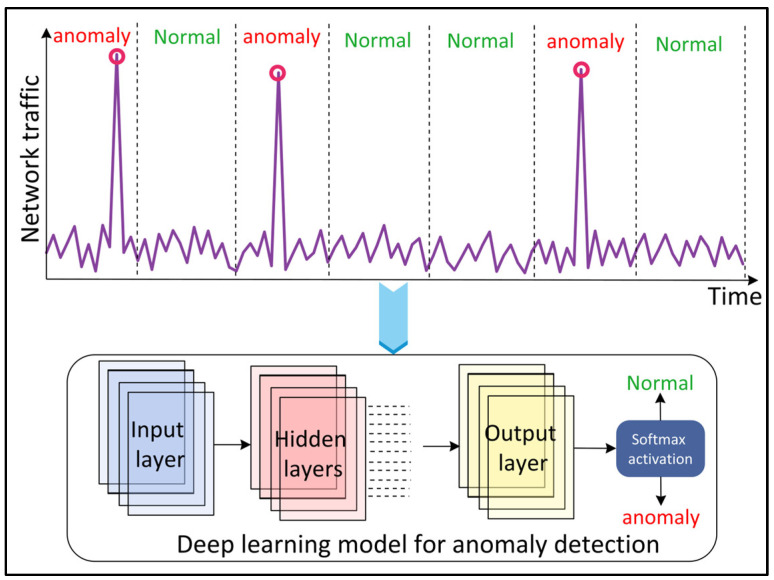
Deep Learning (DL)-based network traffic anomaly detection.

Network traffic anomalies refer to unusual changes in the traffic such as a transient change in users’ requests, port scans, and flash crowds. The detection of such anomalies is important for the security of the network and reliable services. DL approaches have recently gained popularity for traffic anomaly detection in complex, dynamic, and heterogeneous wireless networks (Figure 12). Kim and Cho [69] proposed a C-LSTM neural network to model the spatial-temporal traffic data information and to detect an anomaly. The CNN layer in the model reduces the variation in the information, the LSTM layer models the temporal information, and the DNN layer is used to map the data onto a separable space. The tanh activation function is employed in all the layers except the LSTM output layer, which uses softmax activation. Naseer et al. [70] evaluated the performance of ML and DL models for anomaly detection. The authors implemented extreme learning machine, nearest neighbor, decision tree, random forest, SVM, Naïve Bayes, quadratic discriminant analysis, Multilayer Perceptron (MLP), LSTM, RNN, AE, and CNN models. The results showed that DCNN and LSTM detect anomalies with the highest accuracy.

### 4.13. Botnet Detection

**Figure 13:**
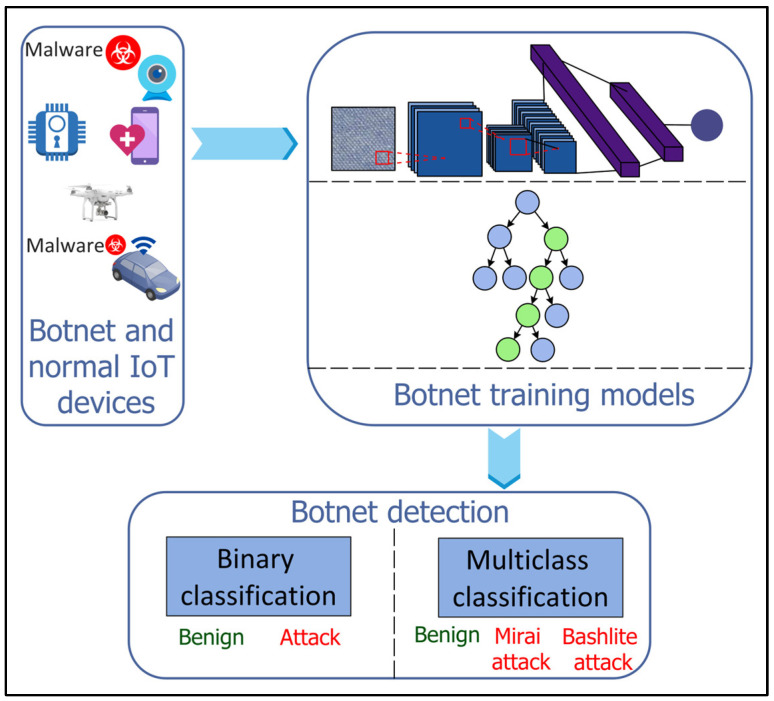
Machine Learning (ML)- and Deep Learning (DL)-based botnet detection for Internet of Things (IoT).

The ever-growing IoT network in smart cities suffers from botnet attacks where a large number of IoT devices are infected by malware to execute repetitive and malicious activities and launch cyber-attacks such as Denial of Service (DoS), distributed DoS (DdoS), or data theft against critical smart city infrastructure [71,72]. For efficient and reliable botnet detection, ML and DL approaches have been restored as potential solutions in the literature (Figure 13). Injadat et al. [73] proposed a combined Bayesian Optimization Gaussian Process (BO-GP) algorithm and Decision Tree (DT) classifier for detecting botnet attacks on the IoT devices. Popoola et al. [74] proposed a DL-based botnet detection system for the resource-constrained IoT devices. The dimensionality of the large volume of network traffic data is reduced using the LSTM autoencoder (LAE) having a Relu activation function and a learning rate of 0.001. A deep Bi-LSTM model, with six input neurons, four dense hidden layers, and an output layer, is then used for botnet detection on the low-dimensional feature set. A Relu activation function is employed in the input and hidden layers, whereas sigmoid and softmax activation functions are used at the output layer for binary and multiclass classification, respectively.

## 5. Taxonomy of Technology-Enabled Smart City Applications in 6G Networks

**Figure 14:**
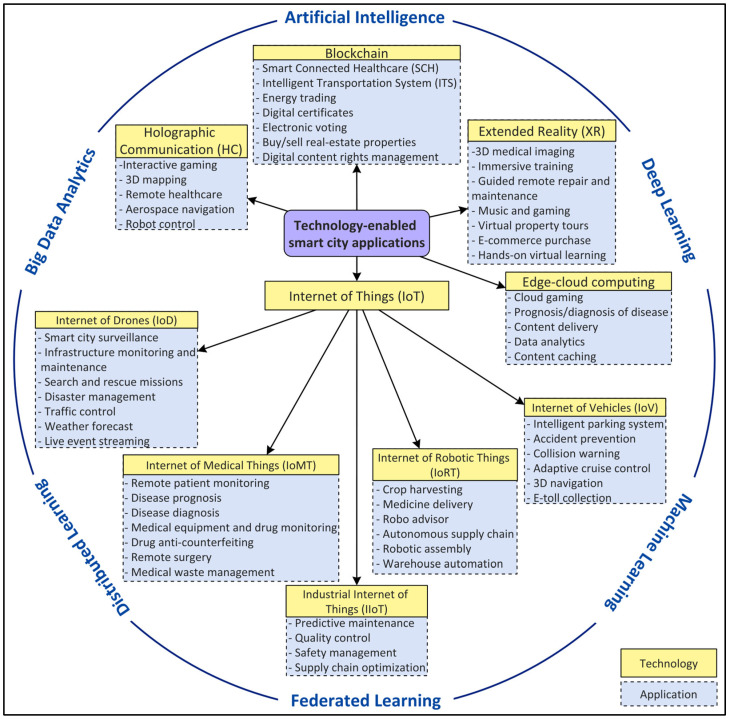
Taxonomy of smart city applications in 6G based on underlying technologies.

In this section, we present a taxonomy of smart city applications for next-generation 6G networks, as shown in Figure 14. We base the taxonomy on the underlying technologies, i.e., IoT, HC, blockchain, XR, and edge-cloud computing, used by those applications empowered by AI, ML/DL, federated and distributed learning, and big data analytics paradigms. In the following, we describe the technologies along with the requirements, in terms of the network characteristics, of the applications using them.

### 5.1. Internet of Things (IoT)

**Figure 15:**
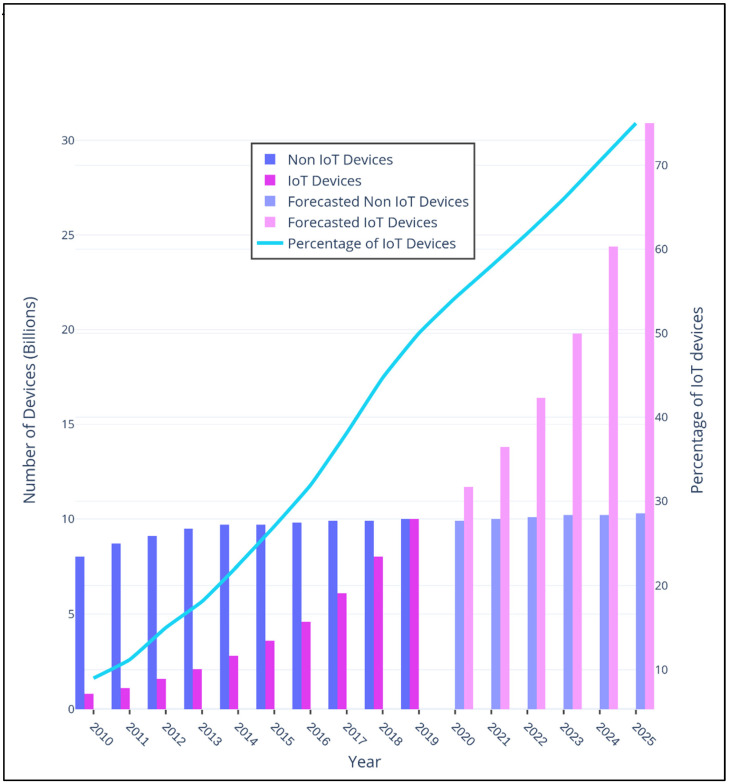
Growth of Internet of Things (IoT) and non-IoT devices from 2010 to 2025.

The IoT is a network of connected devices, sensors, and users using internet technologies that can self-organize, sense and collect data, analyze the stored information, and react to the dynamic environment [75]. The number of connected devices is expected to reach more than 30 billion by 2025 which will be more than 70% of the non-IoT devices. Figure 15 shows the growth of IoT and non-IoT devices over years. The IoT can be further classified based on its application domains such as the IoV, IoMT, IoRT, IoD, and IioT.

#### 5.1.1. Internet of Vehicles (IoV)

**Figure 16:**
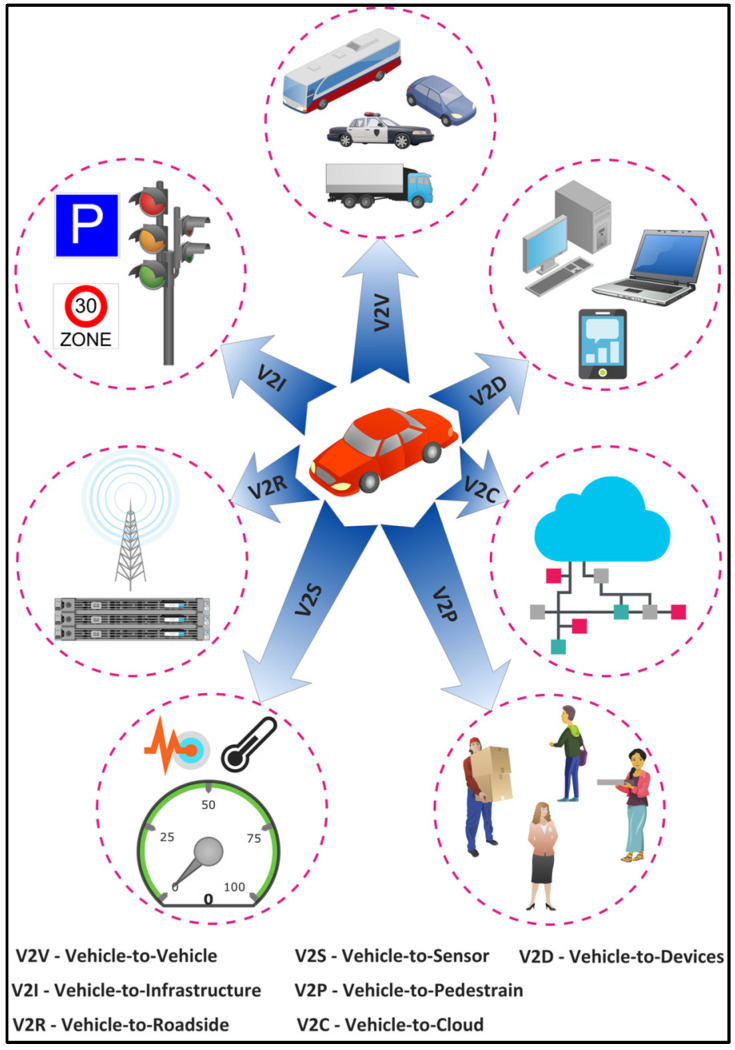
Types of communications on the Internet of Vehicles (IoV).

The IoV is a distributed network of mobile vehicles that have sensing, computing, and Internet Protocol (IP)-based communication capabilities [76]. The global IoV market is projected to reach $208,107 million by 2024 from $66,075 in 2017, with a GAGR of 18% between 2018 and 2024 [77]. The IoV network interconnects vehicles with pedestrian and urban infrastructure facilities such as the cloud and Roadside Units (RSUs). The IoV includes six types of communications for vehicles to receive and transmit data as shown in Figure 16: (1) Vehicle-to-Vehicle (V2V), (2) Vehicle-to-Infrastructure (V2I), (3) Vehicle-to-Roadside (V2R), (4) Vehicle-to-Sensors (V2S), (5) Vehicle-to-Cloud (V2C), and (6) Vehicle-to-Pedestrian (V2P). Several vehicular applications have been developed for the IoV such as an intelligent parking system, real-time navigation, traffic and accident alert, facial recognition for autonomous driving, cooperative adaptive cruise control, and traffic signal violations. These applications have strict data rate and latency requirements that should be supported by the underlying 6G networks. For instance, autonomous driving involving multiple sensors may require a total data rate of 1 Gbps for V2V and V2X communications [78]. Furthermore, it requires a reliability of 99.999% [79], which cannot be obtained with the existing wireless communication systems. In addition, vehicular applications such as infotainment, e-toll collection, collision warning, autocruise, AR map navigation, and co-operative stability control have stringent latency requirements of 500 ms, 200 ms, 100 ms, 20 ms, 5 ms, and 1 ms, respectively [80]. The 6G networks should consider the issues of the limited spectrum, high latency, and low reliability prevailing in the current vehicular standards, i.e., IEEE 802.11p [81].

#### 5.1.2. Internet of Medical Things (IoMT)

The IoMT is a distributed network of bio-medical sensors and devices that acquire, process, and transmit the bio-medical signals of patients. It integrates the communication protocol of the IoT with medical devices to enable remote patient monitoring and treatment. Its global market is expected to reach $172.4 billion by 2030 from $39.3 billion in 2020, at a CAGR of 15.9% from 2021 to 2030 [82]. The IoMT has several applications, such as the monitoring of patients with chronic diseases, monitoring of elderly people, disease prognosis and diagnosis, medical equipment and drug monitoring, drug anti-counterfeiting, and medical waste management. In the context of a pandemic such as COVID-19, the IoMT can be used for the detection, tracking, and monitoring of infected individuals and the prediction of infections [83]. The IoMT applications require ultra-low latency and high reliability for scenarios such as remote surgery. The tactile and haptic internet is the backbone for such scenarios, whose requirements are not completely fulfilled by the current wireless systems [84]. The tactile internet requires an end-to-end latency of the order of 1 ms and haptic feedback requires a latency of sub-milliseconds [85,86].

#### 5.1.3. Internet of Robotic Things (IoRT)

The IoRT is a distributed network of intelligent robot devices that can monitor events, integrate sensors’ data from multiple heterogeneous sources, and use local/distributed intelligence to take actions [87]. The IoRT market is expected to reach $1.44 billion by 2022, growing at a CAGR of 29.7% from 2016 to 2022 [88]. The IoRT has several applications in several domains such as agriculture, construction, logistics, transportation, banking, healthcare, home automation, and industrial automation [89]. Robotics and automation require control in real time to avoid oscillatory movements, with a maximum tolerable latency of 100µs and round-trip times of 1ms [17]. Moreover, industrial robotic automation requires a reliability of 99.999% [79].

#### 5.1.4. Internet of Drones (IoD)

The IoD is a network of coordinated drones with communication capabilities among themselves, pedestrians, and ground infrastructure [90]. The global drone market is expected to reach $43.4 billion by 2027 with a CAGR of 12.56% between 2022 and 2027 [91]. The IoD applications include smart city surveillance, infrastructure monitoring, and maintenance, search, and rescue missions in place of natural/manmade hazards, logistics, traffic control, weather forecasts, disaster management, and events live streaming [92]. These applications require tactile and haptic internet with an ultra-low latency, high data rate, and high reliability requirements.

#### 5.1.5. Industrial Internet of Things (IioT)

The IioT refers to a network of connected machines and devices in the industry for machine-to-machine (M2M) and machine-to-human (M2H) communications [93]. The IioT market is expected to reach $197 billion by 2023 from $115 billion in 2016 with a CAGR of 7.5% from 2017 to 2023 [94]. Applications involve predictive maintenance, quality control, safety management, and supply chain optimization. The IioT sensors and devices are often placed in noisy environments to support mission-critical safety applications. These applications have stringent latency and reliability requirements for proper control decisions [95]. In some cases, the IioT may require a reliability of 99.99999% [96] as information loss could be catastrophic in some scenarios such as nuclear energy plants.

### 5.2. Holographic Communication (HC)

HC is the next evolution of 3D videos and images that will capture data from multiple sources, providing end users with an immersive 3D experience. The global holographic display market size is projected to reach $11.65 billion by 2030 from $1.13 billion in 2020, i.e., a CAGR of 29.1% from 2021 to 2030 [97]. It requires very high data rates and an ultra-low latency. The bandwidth requirements for a human-sized hologram after data compression varies from tens of Mbps to 4.3 Tbps [98,99]. However, a high level of compression to reduce the bandwidth requirements will lead to a high latency. To have a seamless 3D experience, holograms require a latency of sub-milliseconds [17,100]. Consequently, there is a tradeoff between the level of compression, computation bandwidth, and latency, which needs to be optimized by the network [101]. Furthermore, the network should have high resilience in the case of HC to maintain a high QoS by assuring reliability and reducing jitter, packet loss, and latency. Considering the security requirements for HC in applications such as smart healthcare (remote surgery), the network must be secured.

### 5.3. Extended Reality (XR)

Extended reality (XR) technologies involve AR, Mixed Reality (MR), and VR applications. The current wireless communication technologies are unable to provide an immersive XR experience for users of these applications, such as 3D medical imaging, surgical training, immersive gaming, guided remote repair and maintenance, virtual property tours, e-commerce purchase, hands-on virtual learning, and virtual field trips for students. This is due to the inability of the currently deployed 5G technology to deliver ultra-low latencies and very high data rates [102]. These XR applications are highly demanding in terms of communication and computation due to the incorporation of perceptual needs (human senses, physiology, and cognition). The envisioned 6G networks should ensure the Quality-of-Physical-Experience (QoPE) [103] for these XR applications by providing URLLLC and eMBB services.

### 5.4. Blockchain

**Figure 17:**
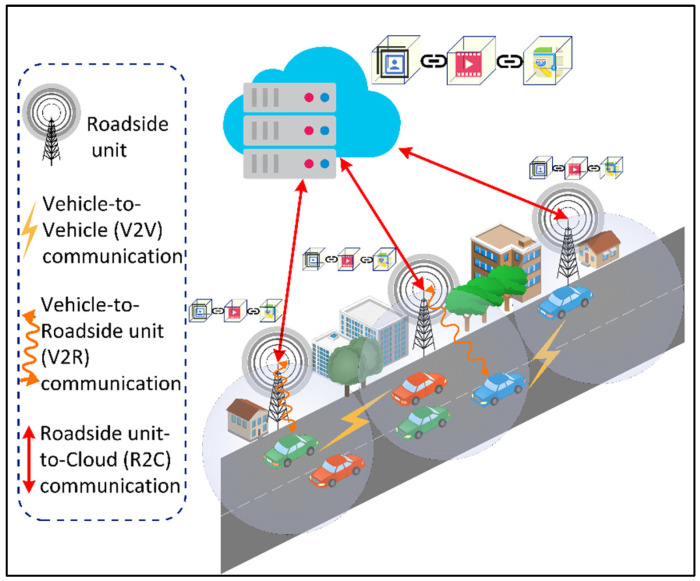
Blockchain-enabled Integrated Internet of Vehicles (IoV)-Edge-Cloud environment.

Blockchain is a decentralized peer-to-peer technology that eliminates the need for a centralized third party [104]. Each event in the network is recorded in a ledger that is replicated and synchronized among all network participants. A participant in the network owns a public–private key pair [105], which enables authentication [106,107] and allows transaction validation. The consensus, cryptographic, provenance, and finality characteristics of blockchain provide security, privacy, immutability, transparency, and traceability. Blockchain has shown potential in several applications including healthcare [108,109,110,111,112], transportation [113,114], energy [115,116], education [117,118], and governance [119,120]. Figure 17 shows a blockchain-based integrated IoV-edge-cloud computing system, where the ledge is replicated at the edge and cloud servers. The events for different vehicular applications such as autonomous driving, infotainment, and real-time navigation are recorded as transactions in the ledger. The consensus protocol and replication of the ledger involved in blockchain require high bandwidth, reliable connection, and low-latency communications between multiple nodes to reduce communication overhead [121,122,123]. It requires a synergistic aggregation of URLLC and mMTC to provide an ultra-low latency, reliability, and scalability.

### 5.5. Edge-Cloud Computing

Cloud computing is a technological paradigm that enables on-demand access to a shared pool of configurable computing, storage, and network resources over the internet [124]. It is based on a pay-per-use model and can be provisioned with minimal management effort. With the emergence of the IoT and big data analytics applications in various domains such as healthcare [125,126,127], education [128,129,130], transportation [131], banking [132,133], energy utilities [134,135], and entertainment [136,137], cloud computing provides a sandbox for data processing and storage, enabling the deployment of compute-intensive smart city applications [138]. However, considering the distance between the IoT devices and the remote cloud servers, the latency requirements of time-critical applications may be violated. Consequently, mobile edge computing has been introduced, which provides computing and storage resources close to the IoT devices. For applications with low latency requirements, the service request can be directed to the nearest edge computing site. However, the computing capabilities of edge servers are low compared to the remote cloud servers, leading to the high processing time for compute-intensive applications. Thus, an integrated edge-cloud computing system is often used to handle compute-intensive and/or time-critical applications [139,140]. However, the underlying 6G networks should consider the energy efficiency [141,142,143,144,145,146,147,148], optimal resource provisioning and scheduling [149,150,151,152], and contextual-aware application partitioning [153,154,155,156,157,158,159,160,161,162,163,164] requirements of this integrated system.

In summary, smart city applications have stringent requirements, in terms of an ultra-low latency, high data rate, reliability, energy efficiency, and security that should be fulfilled by the next-generation 6G networks. These applications, underpinned by emerging technologies such as the IoT, HC, blockchain, XR, and edge-cloud computing, provide a lot of potential for unprecedented services to citizens. However, smart urbanism [165] is seen as critical for the success of a smart city. Governments should put in place plans to address some inherent issues to the deployment of AI-sensing and data-driven smart applications. In particular, privacy concerns should be addressed as personal data is collected continuously. In addition, people’s fear that this paradigm shift to smart cities may generate unemployment in some professions should be dealt with. Furthermore, worries of losing social face-to-face interactions with government entities, which would rely on sensing and digital devices to collect personal data for improving services, have to be taken care of. Consequently, smart urbanism advocates incremental changes to cities rather than a massive one.

## 6. Future Directions

**Figure 18:**
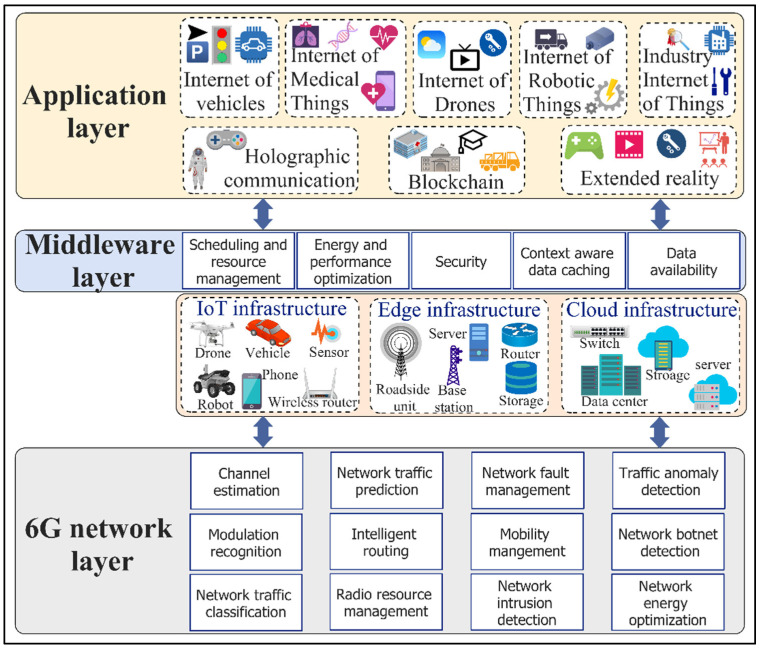
AI-enabled smart city applications in self-learning 6G networks.

While the 5G mobile communication networks generation is just starting to be deployed, there is already a plan to design new 6G networks. This is because of the proliferation of diversified smart city applications that are extensively distributed and more intelligent than ever before, thanks to the emergence of AI, big data analytics, federated and distributed learning, the IoT, edge-cloud computing, and blockchain. The currently in-deployment 5G networks will not be capable of meeting the heterogeneous and stringent requirements of these applications, in terms of efficiency, real-time operation, and reliability, with ever-increasing traffic demands. For instance, 5G is incapable of delivering ultra-low latencies and high data rates for holographic applications that demand data rates of up to 4.3 Tbps. In contrast to the previous generation networks, 6G is expected to support numerously connected and intelligent applications with stringent requirements in terms of high data rates, high energy efficiency, ultra-low latencies, and very broad frequency bands. Considering the requirements of 6G networks and smart city applications, AI will be the dominant enabler in the network, middleware, and application layers, as shown in Figure 18. Current research practices either focus on self-learning 6G networks, AI-enabled middleware, or AI applications in smart cities’ digital ecosystems. AI with self-learning capabilities empowers 6G networks to be intelligent, agile, flexible, and adaptive by providing functionalities for channel estimation, modulation recognition, network traffic classification and prediction, intelligent routing, radio resource management, fault management, network energy optimization, and intrusion, botnet, and traffic anomaly detections. Furthermore, at the middleware layer, AI can aid in the scheduling of smart city applications’ requests, computing resource management, computation and communication energy optimization, application performance optimization, context-aware data caching, and fault tolerance and data availability. In regard to smart city applications, AI can benefit the evolving applications within emerging technological paradigms such as the IoV, IoMT, IoD, IoRT, IIoT, HC, XR, cloud computing, edge-cloud computing, and blockchain.

Research in the following directions is required for the realization of AI-enabled smart city applications in self-learning 6G networks.

1. *Automated AI frameworks:* In 6G networks, a massive amount of the data will be generated from the network, middleware, and application layers. The dynamic environment requires ongoing updates of the AI learning models’ parameters. In 6G networks where ultra-low latencies are a key requirement, tuning the parameters using traditional grid search or meta-heuristic approaches may introduce a computational overhead, degrading the performance of smart city applications and the underlying 6G networks. Consequently, there is a need for automated AI frameworks that would select the optimal models’ parameters based on the contextual applications and network dynamics.
2. *AI frameworks integration:* The self-learning 6G networks in the smart city digital ecosystem will comprise numerous AI models at the network, middleware, and application layers. The output of the learning models from the application and middleware layers should be fed as the input to the learning models at the network layer in a dynamic environment. The high flexibility and scalability of the AI learning frameworks are crucial for supporting a high number of interactions between the learning models at different layers and providing dependable services in real time. Consequently, further research is required on how to integrate dependable, flexible, and scalable learning frameworks for smart city applications in 6G networks.
3. *Performance of AI models:* In 6G networks, meeting the accuracies of the AI models to process high-dimensional dynamic data at the network, middleware, and application layers is crucial. However, these AI models, deep learning and meta-heuristics in particular, have high computational complexity and require a huge amount of time for convergence. This hinders the deployment of applications with ultra-low latency requirements such as robotics and automation, collision warning in the IoV, and AR map navigation. Furthermore, the computationally expensive AI models have a high energy consumption. Consequently, further research on how to design efficient AI approaches to improve computation efficiency and energy consumption is required.

## 7. Summary and Conclusions

The design of agile, flexible, and self-learning 6G networks is envisioned to support emerging distributed, dynamic, and intelligent smart city applications. AI is expected to play an important role in smart city applications as well as the 6G networks. In this paper, we provide a temporal evolution of wireless network generations capturing the technological and application requirements that led to the development of a given network generation over a certain period. In addition, we adopt a holistic approach to providing taxonomies for AI-enabled 6G networks and technology-enabled smart city applications. For the 6G networks, we highlight the employed self-learning models. Furthermore, for the applications use cases, we provide the QoS and SLA requirements that should be considered for the deployment of these applications. Finally, we discussed research directions toward intelligent and integrated computing, communication, coordination, and decision-making smart city digital ecosystems in 6G networks.

## Figures and Tables

**Table 1:** Summary of related surveys.

Work	Approach	Evolution of Wireless Communication Technology	AI-Enabled 6G Networks	Technology-Enabled Applications in 6G
[16]	Top-down	**✓**	**✕**	**✓**
[17]		**✕**	**✕**	**✓**
[18]	Down-Top	**✕**	**✓**	**✕**
[20]		**✕**	**✓**	**✕**
[19]		**✕**	**✓**	**✕**
[21]		**✕**	**✓**	**✕**
This paper	Holistic	**✓**	**✓**	**✓**

**✓** → considered; **✕** → not considered.

**Table 2:** Characteristics of the wireless communication technology from 1G to 6G.

	Network	1G	2G	3G	4G	5G	6G
Features							
**Start**		1970	1980	1998	2000	2010	2020
**Deployment**		1984	1999	2001	2010	2019	2030 *
**Technology**		AMPS, NMT, TACS	GSM, GPRS, EDGE	WCDMA, UMTS	LTE, WiMAX	MIMO, mm Waves	THz communications, VLC
**Frequency**		30 KHz	1.8 GHz	1.6–2 GHz	2–8 GHz	3–30 GHz	95 GHz–3 THz
**Multiplexing**		FDMA	TDMA/CDMA	CDMA	OFDMA	OFDM	OFDM
**Switching**		Circuit	Circuit, packet	Packet	All packet	All packet	All packet
**Core network**		PSTN	PSTN	Packet Network	Internet	Internet	Internet
**Primary services (in addition to previous generations)**		Voice calls	International roaming voice calls, conference calls, SMS, MMS, WAP, WWW, and emails	Video conferencing, GPS	Mobile web access, IP telephony, 3D videos, HD mobile TV	Machine vision, connected cars, smart homes, AR	Tactile and haptic internet, connected autonomous systems, holographic society
**Peak data rate**		NA	50 Kbps (GPRS) 1 Mbps (EDGE)	21 Mbps	100 Mb/s	20 Gb/s	≥1 Tb/s
**Mobility support**		NA	NA	NA	350 km/h	500 km/h	≥1000 km/h
**Latency**		NA	300 ms	100 ms	10 ms	1 ms	10–100 µs
**Network energy efficiency (compared to 4G)**		NA	0.01x	0.1x	1x	≥10x	≥100x
**Spectral efficiency (compared to 4G)**		NA	NA	0.6x	1x	3x	≥15x
**Area traffic capacity**		NA	NA	1 Kbps/m^2^	0.1 Mbps/m^2^	10 Mbps/m^2^	1 Gbps/m^2^
**Connection density (devices/km^2^)**		NA	NA	10^4^	10^5^	10^6^	10^7^

* → Expected; AMPS → Advanced Mobile Phone System; NMT → Nordic Mobile Telephone; TACS → Total Access Communication System; GSM → Global System for Mobile; GPRS → General Packet Radio Service; EDGE → Enhanced Data rates for GSM Evolution; WCDMA → Wideband Code Division Multiple Access; UMTS → Universal Mobile Telecommunications Service; LTE → Long-Term Evolution; WiMAX → Worldwide Interoperability for Microwave Access; MIMO → Multiple Input Multiple Output; THz → Terahertz; VLC → Visible Light Communication; FDMA → Frequency Division Multiple Access; TDMA → Time Division Multiple Access; CDMA → Code Division Multiple Access; OFDMA → Orthogonal Frequency Division Multiple Access; OFDM → Orthogonal Frequency Division Multiplexing; PSTN → Public Switched Telephone Network; SMS → Short Message Service; MMS → Multimedia Message Service; WAP → Wireless Application Protocol; WWW → World Wide Web; GPS → Global Positioning System; HD → High Definition; AR → Augmented Reality; NA → Not Applicable.

## Data Availability

Not applicable.
